# Docendo discimus: promoting patient personhood in clinical supervision – a critical phenomenological study of reciprocal professional formation in medicine

**DOI:** 10.1186/s13010-026-00221-6

**Published:** 2026-06-03

**Authors:** Josephine T. V. Greenbrook, Michael Ioannou, Aliénor H. Lemieux-Cumberlege, Lena Gross

**Affiliations:** 1 School of Public Health and Community Medicine, Institute of Medicine, Sahlgrenska Academy, University of Gothenburg, Gothenburg, Sweden; 2 Mason Institute for Medicine, Life Sciences and the Law, Edinburgh Law School, University of Edinburgh, Edinburgh, UK; 3 Department of General Psychiatry, Sahlgrenska University Hospital, Gothenburg, Sweden; 4 Department of Psychiatry and Neurochemistry, Institute of Neuroscience and Physiology, Sahlgrenska Academy, University of Gothenburg, Gothenburg, Sweden; 5 Grampian Integrated Drug & Alcohol Psychology Service, NHS Grampian, Grampian, UK; 6 Department of Clinical Psychology, School of Health in Social Science, University of Edinburgh, Edinburgh, UK; 7 Department of High North, Norwegian Institute for Cultural Heritage Research, Tromsø, Norway; 8 Centre for Sámi Studies, UiT The Arctic University of Norway, Tromsø, Norway

**Keywords:** Patient personhood, Clinical supervision, Critical phenomenology, Medical education, Professional identity formation, Hidden curriculum, Medical humanism

## Abstract

**Background:**

Medicine has long been understood as both a scientific discipline and a moral and relational practice. Yet within contemporary hospital environments shaped by efficiency demands, digital infrastructures, and performance metrics, the promotion of a patient’s personhood cannot be assumed to remain visible or structurally supported. Clinical supervision represents a central site in which professional values are embodied and transferred to the future generation of physicians, yet little is known about how supervisors themselves experience sustaining their own commitments to personhood within this context.

**Methods:**

This study explores how clinical supervisors experience fostering and sustaining the promotion of patient personhood while supervising medical students, interns, and residents in two Swedish university hospitals. Seventeen physicians participated in in-depth semi-structured interviews. Data analysis employed a critical phenomenological approach, enabling examination of both lived experiences and the institutional conditions shaping these.

**Findings:**

Two interrelated dimensions were identified. First, supervisors described actively rendering patient personhood visible and legitimate within clinical reasoning, translating relational attentiveness into recognised forms of professional competence, modelling humility within hierarchical settings, and protecting core humanistic values within organisational cultures. Second, they described sustaining personhood in their own work as ongoing moral work requiring development over time, reminders in practice, inspiration through interaction, and deliberate inner resolve. Promoting personhood, thus, emerged as reciprocal and cumulative, where through teaching, supervisors simultaneously renewed and reshaped their own ethical commitments.

**Conclusions:**

The findings suggest that patient personhood is not passively inherited within contemporary clinical environments but actively cultivated through supervisory and relational practice. By situating supervisors’ experiences within organisational and cultural structures, the study contributes a theoretically grounded account of how medicine’s dual character as both a technocratic and a humanistic practice is negotiated in everyday work. Ultimately, sustaining patient personhood, and promoting it in the future generation of physicians depends not only on supervisors’ individual commitment but also on institutional conditions that either enable or constrain its visibility.

*“Docendo discimus.”*

*[By teaching, we learn.]*

*– Latin Proverb*

## Introduction

In line with foundational ethical principles in medicine that emphasise respect for autonomy, dignity, and lived experience, promoting patient personhood involves acknowledging that illness is not merely a biological event, but also an existential and relational disruption. Medicine has long been understood as a practice that extends beyond technical competence [1, 2]. Illness unfolds within biography, social context, vulnerability, and meaning, and physicians are expected not only to diagnose and treat disease, but also to recognise and uphold the personhood of those in need of care [3–6]. 

Medicine is both a scientific discipline and a moral and relational practice. Diagnostic precision, technological expertise, and evidence-based reasoning coexist with interpretive judgement, ethical deliberation, and responsiveness to the lived realities of patients. The capacity to move between these dimensions cannot be assumed. It must be cultivated within educational environments that signal what counts as legitimate knowledge and competent practice. Further, whether personhood is treated as integral to medicine or peripheral to it is shaped by how professional norms are modelled and reinforced throughout one’s clinical career [7]. 

Medical education has long sought to foster humanistic values, empathy, and ethical reflection in future physicians [2, 3, 8, 9]. Strategies have included formal ethics teaching, reflective writing, communication training, and bedside supervision [10–13]. Despite these efforts, research continues to describe fragmentation in the teaching and assessment of applied ethics and relational competences [8, 14]. Further, the teaching of reflexivity, and the normalisation of reflexive practice in clinical work remains ephemeral [8, 15]. Ethical instruction has also been argued to remain abstract, separated from clinical decision-making, or positioned as an adjunct rather than as an organising principle of practice [9, 15]. In the midst of this fragmentation, the promotion of personhood risks being rhetorically affirmed while practically marginalised [16, 17]. 

Clinical supervision constitutes a central arena in which professional values are embodied and transmitted. Through observation, participation, and dialogue, medical students learn not only technical skills but also how to orient themselves toward patients [18]. Clinical supervisors shape how students come to understand responsibility, authority, and the moral dimensions of care [19]. Yet, supervisory encounters are rarely neutral. They communicate implicit priorities through tone, pacing, language, and attention. In everyday interactions, supervisors model whether patient personhood is foregrounded, translated into clinical reasoning, or quietly displaced by other demands [20]. Simultaneously, contemporary clinical work unfolds within organisational environments that have undergone significant transformation. Across healthcare systems, reforms oriented toward efficiency, accountability, and standardisation have reshaped hospital practice [21, 22]. Expanded documentation requirements, performance measurement, and economic governance coexist with expectations of high-quality relational care [23]. Clinical tempo has intensified, and physicians frequently navigate competing demands within constrained time frames.

These structural conditions are not inherently opposed to the promotion of personhood. However, they shape the conditions under which it can be enacted and sustained [24–26]. When time is compressed and administrative tasks multiply, attentiveness to the existential and relational dimensions of illness may require deliberate effort. In such contexts, the recognition of personhood may not be automatically embedded in routine practice but instead demand active reaffirmation and fostering. The hidden curriculum is particularly relevant in this regard. Organisational culture, hierarchy, and everyday routines shape what students perceive as valued in clinical environments [27–29]. If efficiency, patient turn-over, or biomedical precision are consistently prioritised, students may internalise these orientations even when formal curricula emphasise ethical attentiveness. Clinical supervisors therefore operate within institutional cultures that both enable and constrain the promotion of personhood.

Reflexive skills are vital in developing capacities for reflexivity in context, deepening the learning and integrating of complex subjects, and shaping both physician and clinical supervisor identity [30–32]. This is of increased importance, as the longitudinal effects of medical ethics training have been noted in the literature, with positive impacts reported as being salient across physicians’ professional lives, and translating into applied reasoning throughout their careers [33–35]. Although previous studies have examined role modelling, empathy development, and professional identity formation [3, 30, 31, 36], fewer have explored how clinical supervisors themselves experience the responsibility of sustaining and transmitting commitments to patient personhood within contemporary institutional contexts. Understanding this requires attention not only to individual meaning-making but also to the organisational and cultural structures within which supervision takes place.

The present study explores how clinical supervisors experience fostering and sustaining the promotion of patient personhood while supervising medical students in the context of everyday practice in university hospitals. By applying a critical phenomenological lens, the study seeks to illuminate how commitments to personhood are enacted, negotiated, and at times defended within the organisational realities of contemporary hospital medicine, including institutional arrangements, normative practices, and professional hierarchies.

## Methods

This study employed a critical phenomenological approach. Phenomenology is concerned with examining lived experience and the meanings individuals attribute to their engagement with the world. Rather than treating experience as isolated or purely subjective, phenomenology understands individuals as situated beings-in-the-world whose perceptions and actions unfold within social, institutional, and historical contexts. Critical phenomenology extends this approach by attending to how lived experience is shaped by broader structures of power, normative practices, and institutional arrangements [37]. It asks not only how individuals experience a phenomenon, but how those experiences are conditioned by the environments within which they are embedded [38, 39]. 

In the context explored in this study, this entails examining how supervisors’ efforts to sustain and model attention to patient personhood are shaped by organisational demands, professional hierarchies, and the structural conditions of contemporary hospital medicine. The aim was therefore not only to describe how clinical supervisors articulate their commitments to patient personhood, but to interpret how these commitments are lived, negotiated, and sometimes strained within institutional contexts. This approach allows for an analysis that remains grounded in participants’ narratives while situating those narratives within the structural realities of contemporary healthcare systems.

### Participants and sampling

All participants were physicians who supervised medical students as part of their everyday practice. Participants were recruited from two major Swedish university hospitals, using publicly available contact information. In total, 500 randomly selected physicians were invited via email to participate, capturing a broad variation of specialisations. Of these, 52 responded positively. Following receiving further information about the study, 17 physicians took part in an in-depth interview.

Participants represented a range of medical specialisations, ages, and genders (see Table 1 for demographic details). Three were senior attending consultants and the remainder were consultants. Ten held doctoral degrees in addition to their medical training, and several occupied academic teaching positions within medical education.

**Table 1:** Participant demographics

Participant	Age Range	Gender	Specialisation
P1	70 >	Male	Paediatric Infectious Disease
P2	45–49	Male	Neonatology
P3	70 >	Male	OBGYN
P4	40–44	Female	Paediatrics
P5	45–49	Female	Paediatric Anaesthesiology
P6	45–49	Male	Intensive Care
P7	40–44	Male	Neurology
P8	45–49	Female	Emergency Medicine
P9	35–39	Male	Anaesthesiology
P10	45–49	Male	Vascular Surgery
P11	55–59	Male	Paediatric Anaesthesiology
P12	35–39	Male	OBGYN
P13	60–64	Female	Urology
P14	55–59	Female	Rehabilitation Medicine
P15	45–49	Female	Anaesthesiology
P16	45–49	Male	Cardiology
P17	45–49	Female	Paediatrics

Note. Average age at time of data collection was 49 (median = 46)

### Data collection

Data were collected through semi-structured, in-depth interviews conducted exclusively by the first author, as part of a broader study that investigated the role of clinical empathy in patient personhood, in physicians' lived experience. Interviews averaged 71 min in duration, generating a total of 21.2 h of recorded material. All participants provided informed consent prior to participation.

The interview guide included open-ended questions exploring broader understandings of personhood in clinical practice, biopsychosocial elements of clinical care, supervisors’ own experiences of clinical education, their approaches to supervising medical students, and perceived challenges and supports in sustaining attention to personhood in their everyday work.

Participants were encouraged to reflect on concrete clinical situations and to describe how they navigated technical, organisational, and relational aspects of practice. This approach enabled access to experience-near accounts while allowing space for unanticipated dimensions to emerge.

### Data analysis

All interviews were transcribed verbatim. Subsequently, analysis proceeded in three iterative stages [40]. First, reduction and immersion involved repeated close reading of transcripts to identify significant experiential statements related to supervising medical students and sustaining commitments to patient personhood. Second, thematic organisation entailed open coding and clustering of experiential patterns into categories reflecting shared structures of meaning. Attention was paid not only to recurrent themes but also to tensions, ambiguities, and moments of strain. Third, critical interpretation involved examining how thematic patterns were shaped by institutional culture, temporal pressures, professional hierarchies, and structural expectations [37, 38]. 

Throughout analysis, attention was given to contextual, relational, and structural dimensions of experience [39]. Particular focus was placed on how supervisors described time constraints, administrative pressures, professional norms, and organisational culture in relation to sustaining ethical commitments to personhood. This interpretive move allowed the study to illuminate how lived moral engagement unfolds within institutional realities.

Reflexive discussions among the interdisciplinary research team supported interpretive depth and mitigated the influence of unexamined assumptions [41, 42]. Combing perspectives from medicine, sociology, psychology, social anthropology, and bioethics, the authors’ diverse disciplinary and personal backgrounds informed analytic dialogue, facilitating awareness of how positionality shaped interpretation.

### Ethical considerations

The present study was conducted in accordance with the BPS Code of Human Research Ethics [43]. Both written and verbal informed consent were obtained prior to each interview. Participation was fully voluntary and confidential, and participants were informed that they could withdraw from the study at any time without consequence. All collected data were anonymised prior to analysis.

Ethical approval was sought and granted prior to data collection by the ethics committee at the School of Psychology at the University of Liverpool as part of JG's dissertation work. In accordance with Swedish ethics regulations, additional regional ethical review was not required for this study. Organisational approval was, however, obtained from both participating hospitals prior to recruitment taking place.

## Findings

**Fig. 1:**
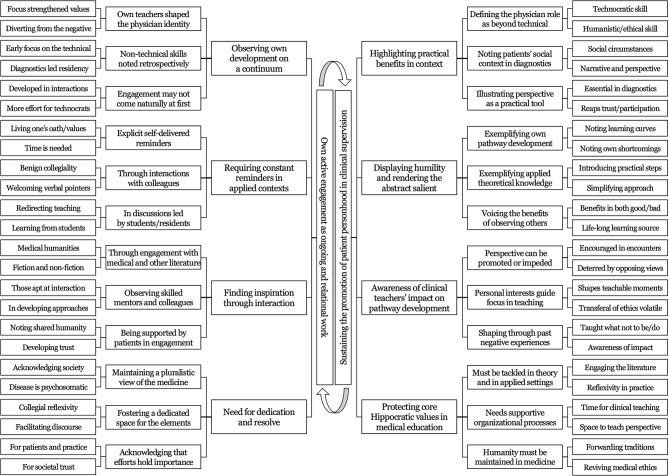
Empirical themes, categories, and subcategories

Analysis revealed a coherent experiential structure underlying supervisors’ accounts of promoting patient personhood in clinical supervision. While participants described diverse clinical contexts and personal trajectories, their narratives converged around a shared pattern of engagement. Figure 1 illustrates the analytic progression from emergent codes to sub-categories, categories, and overarching themes.

Two interrelated dimensions were identified. The first concerns how supervisors render patient personhood visible and legitimate within everyday clinical work. The second concerns the ongoing personal engagement required to sustain this commitment over time. Together, these dimensions demonstrate that promoting personhood in supervision is both contingent upon and reinforcing of supervisors’ own active humanistic engagement.

### Sustaining the promotion of patient personhood in clinical supervision

Supervisors described the promotion of patient personhood in clinical supervision as ongoing work sustained through several interconnected practices: demonstrating its practical clinical value, rendering abstract commitments teachable through modelling and reflection, recognising the formative influence of supervision on professional identity, and protecting these values within broader organisational cultures.

#### Highlighting practical value in context

Across interviews, supervisors expressed a shared conviction that the physician’s role should extend beyond technical expertise. As one participant put it, “A core value of being a good doctor is actually being more than just a technician” (P5). This orientation was not only a personal stance but something supervisors described deliberately foregrounding in their teaching.

Several explained that students initially conceptualise clinical cases primarily in biomedical terms. Supervisors therefore sought to redirect attention toward the lived dimension of illness. One described teaching junior colleagues to recognise that “they are getting medical information also by just talking about regular life” (P6), thereby expanding what counts as clinically relevant knowledge. Another participant illustrated how this shift was enacted in supervision:

Ask the students ‘what is this disease?’ […] The answers they give are almost always biological […] I try to give them that [other] perspective […] You always have to remember that, in there, somewhere, is a person who actually has some symptoms which they live with, and that is completely different from seeing [the disease] from the outside. (P7)

Here, the supervisor explicitly contrasts biomedical abstraction with the embodied and experiential reality of illness. Personhood becomes something that must be reinserted into disease-focused reasoning.

Supervisors also described demonstrating, in their own practice, that attentiveness to personhood has practical benefits. One reflected that “I give it some life and I say this is really working because I know it does […] I have shown that it works also” (P17). Rather than appealing solely to ethical ideals, supervisors often justified recognition of personhood in pragmatic terms, linking it to diagnostic accuracy, communication, and outcomes.

Through a critical phenomenological lens, this repeated need to demonstrate that attention to personhood “works” suggests that it is not automatically recognised as central within dominant clinical logics. Supervisors appeared aware that without being translated into instrumental value, recognition of personhood risks marginalisation in environments where measurable competence and efficiency are highly visible markers of professional success.

#### Displaying humility and rendering the abstract salient

Supervisors described efforts to make commitments to personhood concrete and teachable. Several emphasised that such attentiveness need not depend on extraordinary moral character but could be cultivated through practice. One explained that one can “just put it in a formula […] you should train and you should get it from the beginning […] you’re going to find out that it works” (P4). Another noted that students do not have to be “this perfect super[human],” but can begin by learning “empathic questions” or “empathic lines” that structure interaction (P17). Language itself was described as formative. One participant recalled how a former supervisor “put words to something really important,” noting that while many clinicians “do right things without putting words on them,” articulating them becomes “a good tool, to really improve” (P11). Making personhood explicit through language was thus seen as strengthening its legitimacy and transmissibility.Learning through observation and experimentation was repeatedly emphasised. As one supervisor described it, clinical development unfolds through “lifelong trial and error,” testing what works with one patient and adjusting for the next (P5). Personhood, in this sense, is not a fixed trait but something enacted and refined through practice. Supervisors also described modelling humility by acknowledging their own development. One participant explained, “I do engage, and I realised one of the ways to do that is actually to take yourself, the previous version of yourself, and you criticise it [in front of the medical student]” (P16). By exposing earlier shortcomings, supervisors created space for reflexive dialogue rather than presenting themselves as finished moral authorities.

These accounts suggest that recognition of personhood is often translated into teachable practices within structured and time-pressured environments. While such translation may facilitate transmission, it also risks narrowing the existential depth of personhood to formulaic techniques. At the same time, the modelling of humility can be understood as an attempt to soften hierarchical dynamics and counteract cultures that privilege decisiveness and technical mastery over vulnerability and reflection.

#### Awareness of supervisory impact on professional formation

Many supervisors expressed strong awareness of their formative influence. Reflecting on supervisory models, one explained that “a lot of things about the physician comes from this mentor-mentee relationship,” recalling how, as students, they discussed supervisors’ behaviours and recognised their “huge impact” (P9).

Participants described being shaped by both positive and negative exemplars. Some recalled mentors who were “very empathically and very communicatively” engaged (P8), while others remembered “loads of bad examples” that clarified “how I didn’t want to become” (P6). Through such experiences, supervisors developed their own orientation toward personhood.

They also acknowledged that attention to personhood varies considerably. As one noted, it is “very dependent on the [clinical supervisor], how much they focus on this,” since physicians “are not equally interested in this” (P9). This variability highlights supervision as a site of professional reproduction where commitments to personhood are unevenly transmitted.

This unevenness reflects institutional contexts in which personhood is not consistently reinforced structurally. When recognition of personhood depends primarily on individual supervisors’ interest, its continuity becomes fragile and contingent rather than embedded within organisational norms.

#### Protecting core professional values within organisational culture

Supervisors also described efforts to protect and reinforce commitments to personhood at an institutional level. One argued that “the clinical leadership should promote this, because this is important” (P16), indicating that personhood requires endorsement beyond individual practice. Another emphasised that “culture is so important to forming or shaping your junior colleagues” (P8), highlighting the broader organisational context in which professional values are transmitted. Reflection itself was described as essential. As one supervisor put it, it is “so important to really think about what we’re doing and why we’re here,” because asking “what are we doing here?” can improve practice (P11). Such statements suggest that recognition of personhood is not automatically sustained by institutional structures. Instead, supervisors experience it as something requiring conscious reinforcement within cultures shaped by efficiency, scientific advancement, and administrative demands.

From a critical phenomenological standpoint, the language of protection and promotion indicates that commitments to personhood are lived within systems structured by competing priorities. Sustaining them appears not as passive inheritance but as ongoing ethical work shaped by institutional conditions.

### Own active engagement as ongoing and relational work

Supervisors consistently described sustaining commitments to patient personhood as requiring active and continuous engagement. This commitment was not portrayed as a fixed trait or stable professional achievement, but as something that develops over time, requires reminders, draws on inspiration, and demands deliberate dedication.

#### Development across a professional continuum

Many supervisors reflected on their own formative experiences as medical students and resident physicians. Encounters with mentors had “really put a mark” on them (P11), shaping how they understood what kind of physician they wanted to become. As one participant explained, already as a student one observes clinicians whose patient interactions one wishes to emulate, while others serve as cautionary examples; both teach, because “you reflect” (P9).

Supervisors described their own commitment to personhood as something that matured gradually. One noted that “to be able to engage and actually work with it has come with experience” (P10). Several recalled early career phases dominated by biomedical mastery. One participant explained:

There’s lots of stuff that you can learn from books and the internet, that’s usually the scientific, biological stuff. It’s really easy to get that knowledge, but the other things, that’s hard […] I have had to learn this by doing it. (P7)

Another reflected on the tension between natural inclination and cultivated attentiveness, describing how, for some, focusing on disease and physiology “comes naturally,” while sustaining attention to the human dimensions required effort and years of practice; only after “quite a few years” did they feel they were improving (P2).

These accounts depict recognition of personhood as a developmental trajectory shaped by observation, reflection, and embodied practice rather than as an innate moral quality. From a critical phenomenological perspective, the gradual integration of personhood also reflects how clinical training environments structurally privilege biomedical knowledge early on. Technical expertise is immediately measurable and institutionally reinforced, whereas ethical and relational attentiveness must often be cultivated experientially and over time.

#### The need for constant reminders in practice

Supervisors frequently described the risk of drifting into routinised or “autopilot” modes of practice. One participant observed that one can remain “on autopilot, for years of your career,” only later realising through reflection “how important it is” (P16). Sustaining attention to personhood was therefore described as requiring ongoing reminders in everyday practice, including in clinical supervision.

Collegial observation and feedback were viewed as important supports. One supervisor noted that although feedback is obvious when supervising students, consultants had also discussed the importance of observing one another, often remarking afterward that “we should do this more” (P11). Another described the danger of desensitisation and risks of cynicism settling in over time, explaining that both good and bad habits accumulate, and expressing hope to be reminded of “the bad things” before they become entrenched (P9).

Teaching itself was described as interrupting routine. One participant explained that while attentiveness may occur “every day automatically,” it becomes more deliberate when supervising (P4). Another acknowledged that as human beings one can “sometimes get lazy,” and therefore must “make an effort” to remind oneself and others (P9). Supervisors also described inviting feedback from students, recognising that learners may bring updated knowledge or new perspectives; such exchanges were described as “very meaningful,” reinforcing the sense that “you are never fully trained” (P8).

Taken together, these accounts suggest that recognition of personhood is vulnerable to erosion through repetition and everyday burdens such as workload and stress. The language of autopilot, laziness, and desensitisation reflects the temporal pressures of contemporary clinical practice. Through a critical phenomenological lens, this vulnerability is not simply individual weakness but tied to structural conditions in which efficiency, turn-over, and accumulated workload can narrow attentional focus. Ethical presence, in this sense, must be actively reclaimed rather than passively sustained. Supervision functions not only as pedagogy but as a mechanism of ethical recalibration, and a return to acknowledging patient personhood.

#### Finding inspiration through interaction

Supervisors also described inspiration as essential in sustaining their commitment to patient personhood. Engagement with literature, colleagues, and patients was repeatedly mentioned as revitalising attentiveness. One participant described consuming literature, being influenced by colleagues, and reflecting in patient encounters as a process that makes one “a better doctor,” not necessarily “a better human being,” but more reflective about the human dimensions of care and “influenced by that” (P16).

Observation of colleagues was described as particularly powerful. One supervisor explained:

Seeing others is really helpful. I’ve learned so much by seeing practicing clinicians do their job, and it’s sometimes like magic. […] I love to see my colleagues’ work. Sometimes it’s very difficult with scheduling […] because we’re quite pressed [on time], but I will get small hints about things you can do, and that is really important. (P5)

Patients encountered along the way were also described as teachers. One participant referred to “specific patients who have been willing to teach you, as a future physician, about different things, and willingly shared” (P8).

These reflections suggest that commitments to personhood are sustained not solely through internal resolve but through relational exchange. Inspiration arises in encounters that interrupt routine and reawaken attentiveness. At the same time, the acknowledgment that time pressure makes such observation difficult underscores how institutional tempo shapes access to these sustaining experiences. Inspiration, then, becomes a valued but unevenly available resource within everyday clinical work.

#### Dedication and inner resolve

Finally, supervisors described sustaining commitments to personhood as requiring determination and, at times, inner struggle. One participant articulated this tension vividly:

We do it all day, but there is a difference if you go in and say, ‘hey, wait a minute, now I’m going to do it differently. I’m going to be here and listen.’ […] It’s training, and it’s commitment. It’s kind of a battle you have with your inner self. (P16)

Here, attentiveness is framed as an intentional reorientation rather than an automatic stance. Others expressed determination to shape their clinical supervision and learning environments differently from those they themselves experienced. One supervisor explained a resolve to ensure students “will get a better education than I got,” emphasising that if core organisational values are consistently enacted, they become natural in daily work (P5).

Supervisors also described the personal meaning derived from sustaining this orientation. Engagement with personhood “contributes value for the patients,” but also “contributes value for me” (P14). For some, professional pride depended on this commitment; to be “the best doctor I can be,” one must do this “for myself and for my patients” (P15). Another described it as “incredibly rewarding” to help those “passing through the organisation” develop these skills, noting that while many things are important, this work also matters “for your own person too” (P11).

These accounts portray attention to personhood as both requiring active effort and being meaningful. The metaphor of an inner battle captures the tension between efficiency-driven tasks and intentional presence. Through the critical phenomenological lens, this struggle reflects how institutional structures and professional norms shape habitual action. Commitment to personhood becomes a stance that must sometimes be consciously reclaimed. Simultaneously, supervisors describe this work as reinforcing professional identity and personal coherence, suggesting that sustaining personhood is not only an ethical demand but a source of meaning within contemporary clinical life.

## Discussion

This study explored how clinical supervisors experience fostering and sustaining the promotion of patient personhood while supervising medical students within Swedish university hospitals. By applying a critical phenomenological lens, the analysis moves beyond a descriptive account of motivation and instead situates supervisors’ efforts within the structural, cultural, and hierarchical conditions of contemporary hospital medicine.

The findings suggest that sustaining a commitment to patient personhood is not merely a matter of individual goodwill or professional virtue. Rather, it emerges as ongoing moral and pedagogical work shaped by institutional tempo, professional hierarchies, and organisational culture. Supervisors described actively rendering personhood visible within clinical encounters, translating it into legitimate clinical reasoning, modelling humility within hierarchical settings, and protecting core professional values in environments that do not consistently foreground them.

### Supervisory practice as situated within organisational structures

A central contribution of this study lies in demonstrating how supervisors’ promotion of patient personhood is shaped by the organisational context of hospital work. Participants repeatedly described compressed clinical time, routine pressures, desensitisation, cynicism, and the risk of operating on *autopilot*. Attentiveness to personhood was therefore depicted as something that must be actively reclaimed rather than passively maintained.

Critical phenomenology directs attention to how such experiences are structured. The need to justify attentiveness to personhood in pragmatic terms suggests that dominant clinical logics privilege measurable competence, productivity, and efficiency. When supervisors emphasised that engaging with the patient as a person *works*, they were not only teaching students but negotiating the legitimacy of personhood within prevailing epistemic frameworks. This finding extends existing literature on medical humanism by illustrating how supervisors adapt ethical commitments to fit institutional rationalities rather than simply transmitting them unchanged [13, 44]. 

In this sense, the struggle described by participants reflects not a lack of motivation, but a contextual tension. Patient personhood is not rejected outright, but it competes with organisational priorities that are more visibly reinforced. The accounts illuminate the dual character of medicine as both scientific and human practice. Supervisors did not describe personhood as separate from biomedical excellence, but as a dimension that must be actively integrated into it. In contemporary hospital settings increasingly shaped by digital documentation systems, advanced diagnostics, and data-driven decision-making, this integration is not self-evident. The existential and relational dimensions of illness require deliberate articulation alongside scientific and technological competence.

### Hierarchy, power, and the hidden curriculum

Our findings suggest the presence of an acute awareness among supervisors that medical students observe not only explicit instruction but also everyday conduct. As previous work also suggests [45, 46], supervision thus functions as a site of professional socialisation where norms are reproduced, where learning happens through both positive and negative exemplars. This was also something they showed awareness of regarding their own training. This highlights how medical culture is transmitted across generations through mentor-mentee relationships. At the same time, participants acknowledged that attention to patient personhood varies depending on individual supervisors’ interests. When the transmission of personhood depends heavily on individual disposition, variability and fragmentation emerge.

This variability reflects the operation of the hidden curriculum. Organisational culture and hierarchical dynamics shape what is modelled and what is left implicit [27–29, 47]. In environments where biomedical precision and efficiency are consistently foregrounded, attentiveness to personhood risks becoming secondary unless actively reinforced. Supervisors’ awareness of their influence indicates sensitivity to this process of cultural reproduction.

The modelling of humility described in the findings can also be interpreted in relation to hierarchy. By openly discussing their own development and limitations, supervisors attempt to soften rigid authority structures and create space for reflexive dialogue. Such practices may contribute to psychological safety in supervisory relationships, allowing students to engage more openly with ethical uncertainty. Fostering patient personhood is therefore intertwined with shaping the relational climate of supervision itself.

Supervisors’ emphasis on eliciting patients’ narratives and attending to psychosocial contexts further reflects a commitment to patient agency. Promoting personhood entails recognising patients as participants in care rather than passive recipients of intervention. In modelling dialogical engagement, supervisors contribute to shaping how future physicians understand partnership, responsibility, and shared decision-making within clinical encounters.

### Motivation reconsidered as moral and relational work

Although the original framing of the study emphasised supervisors’ motivation, the findings suggest that motivation alone is insufficient as an explanatory category. Participants did not describe fluctuating willingness to promote personhood. Rather, they described the effort required to sustain such engagement within routine practice.

The language of inner struggle, reminders, desensitisation, and recalibration indicates that sustaining attentiveness to personhood requires ongoing self-regulation. This can be understood as a form of moral labour embedded in everyday work. Supervisors described actively resisting habitual narrowing of attention and consciously reorienting themselves toward listening and presence.

Such accounts resonate with theories of professional identity formation, which emphasise that identity is continuously negotiated within practice rather than acquired once and for all. Supervisors’ reflections suggest that commitment to patient personhood is sustained through repeated enactment in specific situations rather than secured through abstract endorsement. This aligns with perspectives on workplace learning in medical education, where learning is understood as participation in shared practices rather than simple transfer of knowledge [2, 4]. 

By framing motivation as situated moral work rather than static disposition, the study shifts the focus from whether supervisors value personhood to how they maintain that valuation under structural conditions that may not consistently support it.

### Workplace learning and intergenerational transmission

Emblematic of the Latin proverb *docendo discimus*, the findings map the cumulative and reciprocal nature of promoting patient personhood in clinical supervision. Supervisors described supervision as a reciprocal process in which teaching functions as a reminder and corrective for their own practice. Learning and teaching unfold together.

Rather than presenting supervision as a one-directional transmission of values, participants described it as a relational process that reinforces their own attentiveness. Observing colleagues, engaging with literature, and interacting with patients were described as sources of inspiration that interrupt routine and renew commitment, in line with previous literature [2, 48, 49]. These accounts suggest that workplace learning involves both adaptation to institutional norms and selective resistance to them.

Several supervisors described promoting personhood as contributing to professional meaning and pride. In organisational contexts marked by time pressure and administrative demand, maintaining connection to the human dimensions of care may support coherence in professional identity. While this study did not examine burnout directly, the findings suggest that sustained engagement with personhood may function as a resource that counteracts depersonalisation within routine clinical work.

Importantly, the data indicate that promoting patient personhood is not guaranteed by formal curricula alone. It depends on everyday interactions within clinical teams and on organisational cultures that either support or constrain such engagement.

**Fig. 2:**
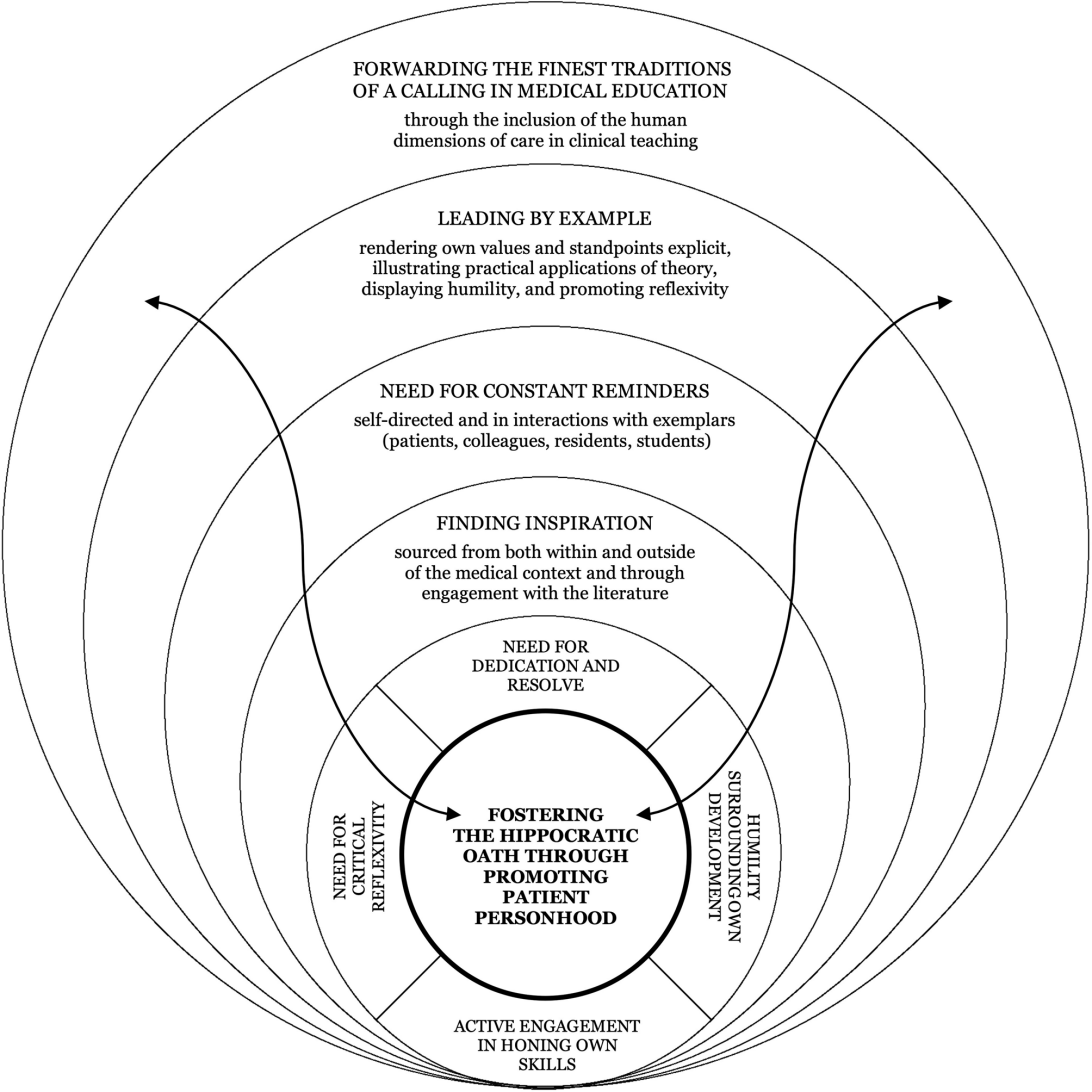
The Reciprocal Professional Formation Model

The reciprocal and cumulative character of this process is synthesised in Fig. 2. The figure conceptualises how promoting patient personhood in clinical supervision simultaneously shapes supervisors’ own professional development. Encounters with patients, colleagues, residents, students, and the literature serve as sources of inspiration and reminders that renew commitment. Through sustained engagement, critical reflexivity, humility regarding their own development, and dedication in practice, the act of forwarding personhood reinforces supervisors’ own ethical orientation. Teaching thus becomes inseparable from ongoing self-formation. The promotion of patient personhood emerges as reciprocal work embedded within everyday clinical practice. 

### Contribution to the field

Previous research has documented tensions between medical humanism and biomedical dominance. The present study adds to this literature by illuminating how these tensions are experienced by clinical supervisors in their everyday work when promoting patient personhood. Rather than reiterating that relational engagement is important, the findings demonstrate how personhood must be actively rendered legitimate, translated into clinical reasoning, and protected within organisational contexts shaped by efficiency demands and institutional priorities.

The study clarifies that the transmission of commitments to patient personhood is structurally uneven. When institutional reinforcement is limited, continuity depends partly on individual supervisors’ engagement. This exposes both the resilience and vulnerability of personhood within contemporary hospital settings. The promotion of personhood is sustained not because structures consistently support it, but because supervisors actively cultivate and defend it within them.

By integrating critical phenomenology, the study moves beyond identifying challenges and instead situates supervisors’ experiences within the organisational, temporal, and hierarchical conditions that shape them. In doing so, it contributes a theoretically grounded account of how the dual character of medicine as both scientific and human practice is negotiated in supervision. It also responds to calls for deeper engagement with humanities and social science perspectives in medical education by demonstrating how institutional culture, power relations, and professional identity formation condition ethical practice.

Ultimately, acute awareness of the fact that development occurs on a continuum throughout one’s medical career is paramount, both for maintaining humility in engagement and as a protective factor in difficult phases. The road toward medical expertise can be demoralizing. A lack of benign guidance and inspiration can give rise to the lures of cynicism and other occupational injuries [50, 51]. When encountering the absence of humility, or somehow sensing a deprivation of shared humanity, explicit efforts must be made to reach outside of mandated supervisory relationships for support. Recognizing and establishing trusting bonds with positive role models who are particularly apt at patient engagement and applied ethics, is critical for revitalizing professional and personal development [45, 49]. Ultimately, medical students and early career physicians should feel encouraged to foster and preserve healthy supervisory relationships, allowing them to morph into longstanding mentorships. In the same vein, senior physicians should feel encouraged to engage in such mentorship, for the benefit of their own development as well.

### Implications

The findings suggest that promoting patient personhood in clinical supervision cannot be understood solely as a matter of individual supervisors’ motivation or ethical orientation. Supervisors described ongoing effort, reminders, recalibration, and at times inner struggle in maintaining attentiveness to the human dimensions of care. This indicates that personhood is not structurally self-sustaining within contemporary hospital environments.

Institutional cultures and organisational structures therefore play a significant role in shaping the conditions under which personhood is transmitted. If efficiency, throughput, and technical mastery are the most visible indicators of professional success, supervisors may feel compelled to frame attentiveness to personhood in instrumental terms in order to render it legitimate. While such framing may secure space for relational engagement, it also risks narrowing personhood to what can be justified through measurable outcomes.

Medical education initiatives aimed at strengthening the promotion of personhood may therefore benefit from moving beyond curricular interventions alone. Communication training and ethics seminars remain important, but the findings indicate that everyday supervisory contexts are central to value transmission. Organisational leadership can influence this process by explicitly recognising engagement with patient personhood as integral to clinical excellence, allocating time for reflective dialogue, and supporting collegial observation and feedback practices.

The modelling of humility described by supervisors also carries implications for hierarchical climate. Supervisory relationships that allow discussion of uncertainty, growth, and ethical ambiguity may contribute to psychological safety and deeper learning. Attention to supervisory development should therefore include relational and reflexive capacities alongside technical teaching skills.

The findings also suggest possible implications for clinician well-being. Several supervisors described sustaining engagement with patient personhood as contributing to professional meaning and coherence. In institutional contexts marked by time pressure and administrative demand, maintaining connection to the human dimensions of care may serve as a counterweight to depersonalisation. While this study did not directly examine burnout, the data indicate that promoting personhood may function not only as an ethical imperative but also as a resource for sustaining professional identity.

Finally, the integration of emerging technologies and digital infrastructures into hospital practice underscores the importance of deliberate engagement with personhood. As documentation systems, decision-support tools, and data-driven processes become increasingly central, the human dimensions of illness cannot be assumed to remain visible. Promoting patient personhood must therefore be actively cultivated within technologically evolving environments rather than treated as an implicit professional inheritance.

### Limitations

Several limitations warrant consideration. Participants were clinical supervisors who volunteered to participate in interviews on a topic concerning patient personhood. It is likely that many held particular interest in relational aspects of care. Their accounts may therefore reflect a level of engagement not uniformly distributed across all clinical supervisors. Perspectives of supervisors who are indifferent or resistant to promoting personhood are not represented. Yet, while random sampling was used for initial contact, variation in professional background and clinical specialty was sought, to capture a breadth of supervisory experiences, allowing exploration of how the phenomenon manifested across different institutional contexts and areas of practice.

Further, the study was conducted in two Swedish university hospitals within a welfare-state healthcare system. Although Sweden maintains formal commitments to equitable and patient-oriented care, organisational pressures related to efficiency and accountability have dominated the past decades. The findings should therefore be understood as situated within specific institutional and cultural conditions. Still, the findings are likely transferrable to other comparable contexts.

Finally, the adoption of a critical phenomenological lens foregrounds structural interpretation. While this perspective enables engagement with hierarchy, organisational culture, and institutional shaping, other theoretical approaches might illuminate additional dimensions of supervisory motivation or learning processes. The present study does not claim theoretical exhaustiveness but offers one interpretive framework that emphasises the interplay between lived experience and structural conditions.

## Conclusion

This study set out to explore how clinical supervisors experience fostering and sustaining the promotion of patient personhood while supervising medical students within Swedish university hospitals. Through a critical phenomenological analysis, the findings demonstrate that commitments to patient personhood are not simply transmitted as stable professional norms. Rather, they are enacted, negotiated, and at times defended within organisational environments shaped by efficiency demands, hierarchy, technological infrastructures, and routine pressures.

Supervisors described rendering patient personhood visible and legitimate within clinical reasoning, modelling humility within hierarchical structures, and actively protecting core professional values in institutional cultures that do not consistently foreground them. Sustaining attentiveness to personhood emerged as ongoing moral and pedagogical work rather than passive adherence to tradition. In line with *docendo discimus*, this work appeared cumulative and reciprocal, as efforts to promote personhood in students simultaneously reinforced supervisors’ own ethical orientation and professional identity.

By foregrounding patient personhood as a lived and negotiated practice within supervision, this study highlights how humanistic commitments in medicine are not simply taught but continually re-enacted within institutional conditions that shape everyday clinical work. More crucially, in situating supervisors’ experiences within the structural realities of contemporary hospital medicine, the study offers a theoretically grounded account of how commitments to personhood are lived in practice, thus moving beyond affirming the importance of medical humanism and instead illuminates the conditions under which patient personhood is maintained, adapted, or placed at risk.

In healthcare systems where scientific innovation, digitalisation, and organisational efficiency continue to expand, the integration of technical expertise with attentiveness to personhood cannot be assumed. It must be deliberately cultivated within supervisory practice and supported at institutional levels. Recognising medicine as both a scientific and human endeavour remains central to sustaining patient personhood within evolving clinical environments.

Ultimately, the transmission of commitments to patient personhood depends not only on individual supervisors’ dedication but also on the organisational contexts that frame their work. Acknowledging this interdependence may be essential for ensuring that future physicians inherit a practice of medicine that honours both its scientific foundations and its responsibility to persons.

## Data Availability

The data analysed during this study are available from the corresponding author upon reasonable request.
